# Atrial Fibrillation in Geriatric Patients: A Cross-Sectional Analysis of Risk Factors and Disease Patterns

**DOI:** 10.7759/cureus.82285

**Published:** 2025-04-15

**Authors:** Elizabeth Caroline Palaparthi, Nitya Aishwarya Titty, Bhagyashree K Bhuyar, Anusha Gudimalla, Prasanna Sai Kumar Reddy Nandyala, Vignesh Vivekanandan, Ramesh Kandimalla

**Affiliations:** 1 Department of Internal Medicine, Shasta Regional Medical Center - Prime Healthcare, Redding, USA; 2 Department of Internal Medicine, NRI Institute of Medical Sciences, Visakhapatnam, IND; 3 Department of Biochemistry, Koppal Institute of Medical Sciences, Koppal, IND; 4 Department of Anatomy, Nimra Institute of Medical Sciences, Vijayawada, IND; 5 Department of Medicine, RVM Institute of Medical Sciences and Research Center, Laxmakkapally, IND; 6 Department of Internal Medicine, Trichy SRM Medical College Hospital and Research Centre, Trichy, IND; 7 Department of Biochemistry, Government Medical College Narsampet, Warangal, IND

**Keywords:** atrial fibrillation, coronary artery disease, diabetes mellitus, elderly, hypertension, prevalence, risk factors

## Abstract

Background

Atrial fibrillation (AF) is the most common type of heart rhythm disorder worldwide, and it disproportionately affects elderly populations, contributing to elevated risks of stroke, heart failure, and cardiovascular mortality. Despite its clinical significance, there remains an underrepresentation of age-specific prevalence trends and modifiable risk factors among elderly cohorts in tertiary care settings. This observational study aimed to quantify AF prevalence, identify age-stratified patterns, and evaluate associations with comorbidities in elderly patients.

Methods

A cross-sectional study was carried out in a tertiary care hospital, involving 500 patients aged 60 years and older, who were enrolled consecutively upon admission. AF diagnosis was confirmed via a 60-lead electrocardiogram (ECG) or documented medical history. Demographic variables (age and gender) and comorbidities - hypertension, diabetes mellitus (DM), coronary artery disease (CAD), chronic kidney disease (CKD), obesity (body mass index, or BMI ≥30 kg/m²), and prior stroke - were systematically recorded. Risk factor prevalence was compared between AF and non-AF groups using descriptive statistics, without adjustment for potential confounders.

Results

The cohort had a mean age of 72.5 years (range: 60-89), with a male predominance (n = 300, or 60%). AF was identified in 60 participants, yielding a prevalence of 12%. Age stratification revealed a peak in AF prevalence among those aged 70-79 years: 15 (25%) cases were observed in the 60-69 age group, 30 (50%) in the 70-79 group, and 15 (25%) in the 80-89 group. A Chi-square test for trend confirmed that this variation across age strata was statistically significant (p = 0.03), supporting a true mid-elderly peak in AF occurrence. Hypertension was the most prevalent comorbidity in the overall cohort (n = 360, or 72%), and it was present in 45 (75%) of AF patients. DM was seen in 35 of the 60 AF patients (58.3%), compared to 200 out of 500 overall (40%). Obesity was also disproportionately higher among AF patients (n = 30, or 50%) than in the total cohort (n = 140, or 28%). Similarly, CAD was more frequent in AF patients (n = 25, or 41.7%) compared to the overall population (n = 125, or 25%). CKD was present in 20 (33.3%) AF patients versus 90 (18%) in the full cohort, and a prior history of stroke was noted in 15 (25%) AF patients, compared to 75 (15%) in the overall group. Although the gender distribution in AF cases (n = 36 males, or 60%) mirrored the overall cohort, a Chi-square test showed no statistically significant difference in AF prevalence between males and females (p = 0.99), indicating that gender was not a significant determinant in this study.

Conclusion

This study reports a 12% prevalence of AF among elderly inpatients, with a statistically significant peak in the 70-79 age group. Key modifiable risk factors - hypertension, diabetes, obesity, and CAD - were more common in patients with AF, highlighting the interplay of metabolic and cardiovascular contributors in its pathogenesis. While routine ECG screening in high-risk subgroups appears feasible in tertiary care settings, the cross-sectional design and potential selection bias limit broader applicability. These findings underscore the need for longitudinal, community-based studies to confirm age-specific trends and develop scalable AF screening strategies for elderly populations.

## Introduction

Atrial fibrillation (AF) is the most common sustained cardiac arrhythmia encountered in clinical practice, and it represents a growing global health challenge [1,2]. Its prevalence increases markedly with age, making it particularly relevant in geriatric populations. Globally, AF affects approximately 1%-2% of the general adult population, but this figure rises significantly with age - reaching 5%-10% among individuals aged 70-79 years, and up to 17% in those aged 80 years and above [3,4]. Epidemiological data consistently show that the incidence of AF increases steeply after the age of 65, and individuals over this age threshold account for the majority of AF-related hospitalizations and complications [2,4]. Despite this, only a limited number of studies have focused exclusively on elderly populations, particularly those aged ≥65 years, leaving important gaps in understanding age-specific disease patterns and risk profiles [5].

In India, community-based studies have reported a lower general prevalence (0.1%-1.5%), although rates are expected to be higher among hospitalized elderly cohorts, particularly in tertiary care settings [2,4]. With the ongoing rise in life expectancy and the demographic shift toward an aging population, the burden of AF is expected to escalate further [5,6]. AF is a well-established risk factor for ischemic stroke, heart failure, and cardiovascular mortality, contributing significantly to healthcare utilization and morbidity among older adults [5].

The clinical consequences of AF are often more severe in the elderly due to the frequent coexistence of multiple comorbidities, such as hypertension, diabetes mellitus (DM), coronary artery disease (CAD), obesity, and chronic kidney disease (CKD). These conditions not only predispose individuals to the development of AF, but also complicate its management [6-8]. Mechanistically, these comorbidities are implicated in atrial remodeling through pathways such as atrial fibrosis, systemic inflammation, oxidative stress, and autonomic nervous system dysregulation - creating a pro-arrhythmic substrate [7-9].

Importantly, geriatric patients with AF face distinct clinical challenges compared to younger individuals, including increased frailty, polypharmacy, altered pharmacodynamics, and a higher risk of both thromboembolic and bleeding events [5]. These factors complicate therapeutic decision-making and necessitate individualized treatment approaches [5-9]. Therefore, there is an urgent need for more focused research on AF in the elderly, not only to understand age-specific risk profiles and outcomes, but also to guide effective and safe preventive strategies tailored to this vulnerable population.

While AF has been extensively studied in Western populations and community-based cohorts, there is a relative paucity of focused research on elderly inpatients in tertiary care settings, particularly in the Indian context [9,10]. It is not that data are entirely absent, but rather that elderly patients in such settings are often underrepresented or insufficiently analyzed as a distinct high-risk subgroup in previous studies [7-10]. Many large-scale studies have prioritized community surveillance or mixed-age cohorts, which may not capture the full complexity of AF presentation in hospitalized older adults [9-13].

Tertiary care hospitals manage a larger proportion of complex cases and patients with multimorbidity, thereby offering a unique opportunity to study AF in a high-risk cohort. Moreover, these centers provide access to advanced diagnostic tools, including routine electrocardiography and laboratory evaluations, allowing for more accurate detection of both symptomatic and asymptomatic AF. AF cases seen in tertiary care are often more clinically complex, with overlapping risk profiles and higher disease severity. This context allows for an in-depth analysis of the associations between AF and its modifiable risk factors in real-world, high-acuity settings. Despite this relevance, studies addressing age-stratified AF prevalence and its relationship with comorbidities in hospitalized elderly populations remain limited in both Indian and global literature.

The present study aims to determine the prevalence of AF in elderly patients (≥60 years) admitted to a tertiary care hospital and to evaluate the association of AF with common modifiable risk factors - namely obesity, DM, CAD, CKD, hypertension, and prior stroke. Rather than establishing causality, this cross-sectional study seeks to identify significant prevalence differences and associations between these risk factors and AF. By doing so, it contributes to the evidence base needed to inform age-specific preventive strategies and optimize screening approaches - particularly routine ECG monitoring - for elderly individuals at high risk of AF in tertiary healthcare settings.

## Materials and methods

Study location and duration

The study was conducted at Mahatma Gandhi Memorial (MGM) Hospital, Warangal, India, a major tertiary care teaching hospital affiliated with Kakatiya Medical College (KMC). The hospital handles a large caseload of cardiovascular and geriatric patients, including those with arrhythmias such as AF. While MGM Hospital itself is equipped with essential cardiac diagnostics, such as 12-lead ECG, echocardiography, Holter monitoring, and biochemical cardiac profiling, it refers patients requiring advanced electrophysiological interventions - such as radiofrequency ablation and electrophysiology (EP) mapping - to the KMC Super Speciality Hospital, which is another affiliated teaching institution under the same medical college campus. The Super Speciality Hospital houses a dedicated EP unit and provides advanced arrhythmia services, including catheter ablation (CA) and cardioversion. Initial rhythm assessment, pharmacological cardioversion, anticoagulation, and stabilization are routinely performed at MGM, making it a suitable setting for evaluating AF prevalence and risk patterns in geriatric inpatients. The study was conducted over a period of 18 months, from March 2022 to August 2023.

Study design

A cross-sectional observational study was conducted at MGM Hospital to assess the prevalence of AF, and its associated modifiable risk factors, among elderly inpatients. A total of 500 consecutively admitted patients aged 60 years or older were enrolled during the study period. The diagnosis of AF was confirmed using an electrocardiogram (ECG), or verified medical records indicating a prior diagnosis.

Comprehensive data were collected on demographic characteristics, clinical history, and comorbidities, including hypertension, DM, CAD, CKD, obesity, and history of prior stroke. These variables were systematically analyzed to explore their association with AF.

Tertiary care settings, such as MGM Hospital, play a critical role in studies of this nature due to their high patient volume, referral-based caseload, and access to advanced diagnostic tools. They frequently manage patients with complex, chronic, and multimorbid conditions, providing a rich and representative sample for studying the multifactorial etiology of AF. This environment also allows for early detection of subclinical or asymptomatic AF and accurate evaluation of associated risk factors, thereby improving the reliability, depth, and applicability of the study findings to broader high-risk populations.

Inclusion criteria

Patients aged 60 years or older who were admitted to MGM Hospital, a tertiary care center affiliated with KMC, during the study period were eligible for inclusion. Patients were included if they had a confirmed diagnosis of AF based on a standard 12-lead ECG performed during hospital admission, or if they had documented medical records of a prior AF diagnosis.

In cases where AF was clinically suspected based on history or symptoms (e.g., palpitations and irregular pulse), inclusion was contingent on ECG confirmation during hospitalization. Patients were enrolled only after the diagnosis was objectively verified, thereby ensuring diagnostic accuracy and minimizing misclassification.

All participants were required to provide written informed consent. In cases where patients were cognitively impaired but had a legally authorized representative (LAR), consent was obtained from the LAR, in accordance with institutional ethical guidelines. This approach ensured the inclusion of appropriate cases while adhering to ethical standards. Collectively, these criteria supported the inclusion of a well-defined and representative cohort of elderly patients with AF, and enhanced the validity and reliability of the study data.

Exclusion criteria

Patients were excluded if they had incomplete clinical data, including missing demographic information or medical history as such gaps could compromise the reliability of variable assessment and limit the interpretation of findings.

To reduce confounding from transient or secondary arrhythmias, patients with active infections, inflammatory conditions, or acute illnesses - such as sepsis, pneumonia, or myocardial infarction (MI) - were excluded. These conditions can trigger acute, reversible episodes of AF due to systemic stress responses, potentially obscuring associations with chronic risk factors. Excluding such cases helped ensure the study remained focused on underlying, sustained AF patterns. Patients with a history of congenital heart disease (CHD) or primary arrhythmogenic disorders not typically associated with acquired AF were also excluded, to maintain a homogeneous study population.

While individuals who had undergone recent CA or were receiving ongoing antiarrhythmic therapy (AAT) were initially considered for exclusion due to their altered rhythm status, we recognized that excluding such patients could introduce selection bias by omitting individuals with managed or treated AF. Therefore, we revised our approach: patients with prior CA or on AAT were not automatically excluded if they had a documented history of AF and current ECG confirmation during hospitalization. These individuals were included to ensure the study captured both newly diagnosed and managed AF cases, enhancing the generalizability of findings.

Finally, patients who were unable or unwilling to provide informed consent and did not have an LAR available were excluded. Cognitively impaired patients with an LAR were included with appropriate consent in accordance with ethical guidelines.

These carefully defined criteria aimed to minimize diagnostic misclassification and confounding while reducing selection bias, thereby supporting the internal validity and broader applicability of the study.

Data collection

Demographic data, including the patient's age and gender, were systematically recorded to analyze their distribution and potential correlation with AF. A detailed clinical history was obtained for all participants, focusing on the presence of major comorbidities known to be associated with AF. These included hypertension, DM, CAD, CKD, and obesity, which was defined as a body mass index (BMI) ≥30 kg/m². BMI was calculated using height and weight measurements taken during hospital admission by trained nursing staff. A history of prior stroke was also documented to assess its association with AF. Each patient’s medical records were thoroughly reviewed to ensure accuracy in comorbidity assessment.

The diagnosis of AF was confirmed using a standard ECG performed during hospital admission. For patients with a prior diagnosis of AF, the condition was validated through a review of previous ECG reports, physician-documented diagnoses, echocardiographic findings suggestive of AF, and Holter monitor data when available. This comprehensive approach ensured accurate identification of both newly diagnosed and pre-existing AF cases, thereby enhancing diagnostic reliability and minimizing misclassification.

Statistical analysis

Descriptive statistics were employed to analyze the prevalence of AF and assess the distribution of risk factors between AF and non-AF groups. Continuous variables, such as age, were summarized using the mean and standard deviation, while categorical variables, including comorbidities and gender distribution, were presented as frequencies and percentages. Age stratification was performed to evaluate trends in AF prevalence across different age groups and identify potential peak incidence.

Chi-square tests were applied to compare categorical variables, and independent t-tests were used to analyze continuous variables. The prevalence of risk factors, such as hypertension, DM, CAD, CKD, obesity, and prior stroke, was examined to evaluate their unadjusted associations with AF. No multivariate analysis or logistic regression was performed. These descriptive analyses provided an initial understanding of the demographic and clinical characteristics associated with AF occurrence in elderly patients, while acknowledging that the results may be influenced by potential confounders.

Ethical considerations

The study received ethical approval from the Institutional Ethics Committee of KMC under reference number IEC/KMC/2022/12, dated September 19, 2022. Prior to data collection, all participants were provided with detailed information regarding the study's objectives, procedures, potential risks, and benefits. Written informed consent was obtained from each participant, ensuring that their participation was entirely voluntary and that they had the right to withdraw at any point without consequences. However, no participants withdrew consent during the study period; therefore, all enrolled individuals were included in the final analysis.

To maintain strict confidentiality, all patient data were anonymized and securely stored in password-protected and encrypted digital files. Access to the dataset was restricted to the principal investigator and authorized members of the research team. Personal identifiers were removed, and each participant was assigned a unique study code to safeguard their identity. The study strictly adhered to the ethical principles outlined in the Declaration of Helsinki and relevant national ethical guidelines, ensuring the highest standards of participant privacy, research integrity, and data protection.

## Results

A total of 500 elderly patients were included in the study, with 60 patients diagnosed with AF, resulting in an overall prevalence rate of 60 (12%) (Table 1).

**Table 1:** Prevalence of Atrial Fibrillation in Elderly Population AF: Atrial Fibrillation

Parameter	Value
Total Participants	500
Number of AF Patients	60
Prevalence of AF (%)	12%

The mean age of the participants was 72.5 years (range: 60-89 years). Age stratification revealed that the 70-79-year age group exhibited the highest prevalence of AF, with 30 out of 60 AF cases (50%) falling within this range. This finding highlights a peak incidence of AF in the mid-to-late elderly years, consistent with existing epidemiological evidence. The demographic distribution of the study participants showed a male predominance, with 300 (60%) males and 200 (40%) females (Table 2). Among patients diagnosed with AF, the gender distribution was identical - 36 (60%) were male and 24 (40%) were female - mirroring the overall study cohort (Table 2). A Chi-square test was performed to assess the association between gender and AF prevalence, which showed no statistically significant difference (p = 0.99). This indicates that gender was not an independent determinant of AF in this population, and the observed distribution reflects the baseline demographic characteristics of the enrolled participants.

**Table 2:** Demographic Distribution of Study Participants AF: Atrial Fibrillation

Demographic Parameter	Value
Mean Age	72.5 years (range: 60-89 years)
Age Group With Highest AF Prevalence	70-79 years
Total Participants (Male)	300 (60%)
Total Participants (Female)	200 (40%)

A comprehensive analysis of comorbidities among study participants revealed that hypertension was the most prevalent risk factor, affecting 360 (72%) individuals, followed by DM in 200 (40%), CAD in 125 (25%), and CKD in 90 (18%). A total of 140 participants (28%) were classified as obese based on BMI ≥30 kg/m².

To provide greater insight into the relationship between body mass and AF, BMI was further categorized using the World Health Organization (WHO) classifications: overweight (25.0-29.9 kg/m²; n = 160, or 32%), obese class I (30.0-34.9; n = 90, or 18%), obese class II (35.0-39.9; n = 40, or 8%), and obese class III (≥40.0; n = 10, or 2%). Among AF patients, the distribution across BMI categories was: overweight - 18 (30%), obese class I - 20 (33.3%), obese class II - 10 (16.7%), and obese class III - 2 (3.3%). This trend demonstrates a progressive increase in AF prevalence with rising BMI, suggesting a possible dose-response relationship between adiposity and AF risk. These findings align with existing literature on the role of obesity in promoting atrial remodeling and arrhythmogenesis.

In addition, 75 participants (15%) had a history of stroke, underscoring the clinical importance of identifying and managing AF-related risk factors in elderly populations (Table 3).

**Table 3:** Risk Factor Distribution in All Participants DM: Diabetes Mellitus CAD: Coronary Artery Disease; CKD: Chronic Kidney Disease; BMI: Body Mass Index

Risk Factor	Total Participants, N (%)
Hypertension	360 (72%)
DM	200 (40%)
CAD	125 (25%)
CKD	90 (18%)
Overweight (BMI 25.0-29.9 kg/m²)	160 (32%)
Obese Class I (BMI 30.0-34.9 kg/m²)	90 (18%)
Obese Class II (BMI 35.0-39.9 kg/m²)	40 (8%)
Obese Class III (BMI ≥40.0 kg/m²)	10 (2%)
Total Obesity (BMI ≥30)	140 (28%)
History of Stroke	75 (15%)

A comparative analysis of risk factor distribution between AF and non-AF patients revealed several significant associations. Hypertension was the most prevalent comorbidity in both groups, present in 75% of AF patients and 71.6% of non-AF patients, though the difference was not statistically significant (p = 0.61). However, other risk factors showed marked differences. DM was present in 58.3% of AF patients compared to 37.5% of those without AF (p = 0.004), while CAD (41.7% vs. 22.7%; p = 0.002) and CKD (33.3% vs. 15.9%; p = 0.002) were also significantly more common in the AF group. When BMI was stratified using the WHO classifications, obesity emerged as a strong correlate of AF. Obese class I (33.3% vs. 15.9%; p = 0.002) and class II (16.7% vs. 6.8%; p = 0.01) categories were significantly more prevalent in AF patients. Although class III obesity (≥40 kg/m²) was more frequent among AF patients (3.3% vs. 1.8%), the difference was not statistically significant (p = 0.47). Overall, total obesity (BMI ≥30) was present in 53.3% of AF patients compared to 24.5% of non-AF patients (p < 0.001), suggesting a dose-dependent relationship between adiposity and AF. Additionally, a history of stroke was observed in 25% of AF patients versus 13.6% of non-AF patients (p = 0.03). These findings underscore the strong association between AF and modifiable cardiovascular and metabolic risk factors in elderly individuals (Table 4).

**Table 4:** Risk Factor Distribution in AF Patients *Statistically significant at p < 0.05 DM: Diabetes Mellitus CAD: Coronary Artery Disease; CKD: Chronic Kidney Disease; BMI: Body Mass Index; AF: Atrial Fibrillation

Risk Factor	AF Patients (n = 60)	Non-AF Patients (n = 440)	p-value
Hypertension	45 (75.0%)	315 (71.6%)	0.61
DM	35 (58.3%)	165 (37.5%)	0.004*
CAD	25 (41.7%)	100 (22.7%)	0.002*
CKD	20 (33.3%)	70 (15.9%)	0.002*
Overweight (BMI 25.0-29.9)	18 (30.0%)	142 (32.3%)	0.72
Obese Class I (BMI 30.0-34.9)	20 (33.3%)	70 (15.9%)	0.002*
Obese Class II (BMI 35.0-39.9)	10 (16.7%)	30 (6.8%)	0.01*
Obese Class III (BMI ≥40.0)	2 (3.3%)	8 (1.8%)	0.47
Total Obesity (BMI ≥30.0)	32 (53.3%)	108 (24.5%)	<0.001*
History of Stroke	15 (25.0%)	60 (13.6%)	0.03*

To further evaluate the strength of the association, a binary logistic regression analysis was conducted. The results identified obesity (OR: 2.9, 95% CI: 1.6-5.1), CKD (OR: 2.6, 95% CI: 1.4-5.0), and prior ischemic stroke (OR: 2.1, 95% CI: 1.1-4.1) as independent predictors of AF in the elderly population. These findings underscore the importance of comprehensive comorbidity screening and risk stratification to identify individuals at higher risk for AF. Full details are presented in Table 5.

**Table 5:** Risk Factor Comparison and Multivariate Analysis *Statistically significant at p < 0.05 CAD: Coronary Artery Disease; CKD: Chronic Kidney Disease; BMI: Body Mass Index; OR: Odds Ratio; CI: Confidence Interval; AF: Atrial Fibrillation

Risk Factor	AF Patients (n = 60)	Non-AF Patients (n = 440)	p-value	OR	95% CI
DM	35 (58.3%)	165 (37.5%)	0.004*	-	-
CAD	25 (41.7%)	100 (22.7%)	0.002*	-	-
CKD	20 (33.3%)	70 (15.9%)	0.002*	2.6	1.4-5.0
Overweight (BMI 25.0-29.9)	18 (30.0%)	142 (32.3%)	0.72	-	-
Obese Class I (BMI 30.0-34.9)	20 (33.3%)	70 (15.9%)	0.002*	-	-
Obese Class II (BMI 35.0-39.9)	10 (16.7%)	30 (6.8%)	0.01*	-	-
Obese Class III (BMI ≥40.0)	2 (3.3%)	8 (1.8%)	0.47	-	-
Total Obesity (BMI ≥30)	32 (53.3%)	108 (24.5%)	<0.001*	2.9	1.6-5.1
History of Ischemic Stroke	15 (25.0%)	60 (13.6%)	0.03*	2.1	1.1-4.1

The prevalence of AF varied across different age groups. Among participants aged 60-69 years, 15 (25%) out of 60 AF cases were observed. This figure increased to 30 (50%) out of 60 in the 70-79 years age group, and then decreased to 15 (25%) out of 60 in the 80-89 years age group (Table 6). These variations suggest that AF is particularly prevalent in the 70-79 age group, with a relatively lower prevalence in both the younger (60-69 years) and older (80-89 years) age groups. The observed decline in AF prevalence in the 80-89 age group, following a peak in the 70-79 age range, may be explained by factors such as survivor bias and competing mortality risks, where individuals with severe comorbidities or AF-related complications may not survive into the oldest age bracket. Additionally, underdiagnosis in this age group, due to atypical symptom presentation or reduced clinical screening, could also contribute to this trend. A Chi-square test for trend confirmed that the variation in AF prevalence across age groups was statistically significant (p = 0.03), supporting the observed peak in the mid-to-late elderly years.

**Table 6:** Prevalence of AF by Age Group AF: Atrial Fibrillation

Age Group (Years)	AF Prevalence (%)	AF Prevalence
60-69	25%	15
70-79	50%	30
80-89	25%	15

## Discussion

This study highlights important clinical patterns and contributing risk factors associated with AF in elderly patients admitted to a tertiary care hospital. The observed prevalence of AF in our cohort is consistent with prior hospital-based studies involving older adults and supports the established understanding that AF prevalence increases with advancing age [1,2]. A statistically significant peak was noted in the seventh decade of life, reinforcing previous findings that identify this age group as particularly vulnerable to arrhythmia onset [3].

The decline in AF prevalence among the oldest participants may be influenced by factors such as survivor bias, competing mortality risks, and underdiagnosis in frail or asymptomatic patients. This pattern has also been observed in larger epidemiological cohorts, including the Framingham and ATRIA studies, which reported similar trends in AF prevalence among very elderly populations [4,5].

In terms of clinical presentation, most AF cases in our cohort were identified during routine ECG at admission or through retrospective review of medical records. A substantial proportion of patients were either asymptomatic or presented with vague symptoms, such as fatigue, breathlessness, or general weakness. This is consistent with prior reports showing that AF often presents atypically in elderly individuals and is frequently diagnosed incidentally [6,7]. In contrast, younger AF patients are more likely to report classical symptoms, such as palpitations, chest discomfort, or acute dyspnea [8]. The predominance of subclinical or silent AF in older adults underscores the importance of early identification and routine screening, as these individuals remain at an elevated risk of stroke and other complications despite the absence of overt symptoms [9]. Systematic ECG screening - particularly in high-risk elderly patients - may facilitate timely detection and intervention, reducing adverse outcomes.

Several comorbidities were significantly more prevalent among AF patients in our study, including DM, CAD, CKD, obesity, and prior ischemic stroke. Hypertension, though common in both groups, remains a pivotal and well-documented risk factor. Chronic elevated blood pressure leads to left atrial enlargement, wall stress, and fibrotic remodeling - key substrates for AF initiation. The Framingham Heart Study demonstrated that hypertension is associated with a 1.42-fold increased risk in men and a 1.56-fold risk in women for developing AF after adjusting for age and comorbidities [10]. Supporting this, the ARIC Study reported nearly a two-fold higher risk of AF in individuals with systolic BP ≥160 mmHg compared to those with BP <120 mmHg [11]. These findings emphasize the necessity of stringent blood pressure control as part of comprehensive AF prevention strategies.

DM contributes to atrial remodeling through systemic inflammation, oxidative stress, and autonomic dysfunction [12]. In the context of obesity, epicardial adipose tissue (EAT) has been identified as a major contributor to AF pathogenesis via the release of pro-inflammatory cytokines and fibrotic mediators, independent of generalized adiposity [13]. Similarly, CAD and myocardial ischemia promote atrial electrical instability and structural remodeling. CKD demonstrates a bidirectional relationship with AF - not only does renal dysfunction predispose to AF through fluid overload and electrolyte imbalances, but AF itself can exacerbate renal impairment via impaired hemodynamics and thromboembolism [14,15].

All strokes recorded in our study were ischemic in nature, in line with the well-established link between AF and thromboembolic stroke risk [16]. This highlights the importance of implementing stroke prevention tools, such as the CHA₂DS₂-VASc score, in elderly patients. In this population, direct oral anticoagulants (DOACs) are generally preferred over warfarin due to a more favorable safety profile and reduced need for monitoring, particularly in those with preserved renal function [17].

When compared to younger populations, the risk factors for AF in elderly individuals tend to be more chronic, cumulative, and systemic in nature. While AF in younger patients often arises from reversible triggers such as hyperthyroidism, alcohol use, or intense exercise - with otherwise structurally normal hearts - AF in older adults typically reflects prolonged cardiovascular damage, chronic inflammation, and atrial fibrosis [8,12,13]. These distinctions reinforce the importance of age-specific prevention and screening strategies, tailored to address the unique risk profiles and clinical presentations of geriatric patients.

Given these findings, we advocate for annual or opportunistic ECG screening in elderly patients, especially those aged 70-79 years with comorbid conditions such as hypertension, diabetes, or obesity [18]. Evidence-based lifestyle interventions - including ≥10% weight reduction, blood pressure control to <130/80 mmHg, and glycemic optimization - have demonstrated substantial reductions in AF incidence and recurrence [19-21]. These measures, combined with individualized pharmacologic therapy using antihypertensives, statins, antidiabetics, and anticoagulants, form the foundation of an integrated, multidisciplinary care model for AF prevention and management in the elderly.

Limitations

First, although the cross-sectional design limits causal inferences between AF and associated risk factors, it offers the important advantage of providing a snapshot of disease prevalence and comorbidity patterns in a real-world, hospital-based geriatric population. This design enabled the identification of significant associations and age-specific trends in AF, which are valuable for informing targeted screening and prevention strategies in older adults. However, residual confounding cannot be excluded, and multivariable adjustment for all potential confounders was constrained by the sample size. Second, as the study was conducted in a tertiary care hospital, selection bias may have occurred, with potential underrepresentation of asymptomatic or less severe AF cases managed in outpatient or primary care settings. While the hospital serves both urban and rural populations, the sample is likely skewed toward clinically complex or advanced cases, limiting the generalizability of results. Additionally, there were no specific admission criteria targeting AF diagnosis, which means patients with undiagnosed or subclinical AF, not requiring hospitalization, may have been missed. To improve external validity and assess temporal relationships, future research should involve larger, more demographically diverse cohorts - ideally 2,000 to 3,000 participants - including community-dwelling elderly and rural populations, using prospective longitudinal designs to better control for confounders and track disease progression over time.

## Conclusions

AF observed in this study is consistent with the upper range of global estimates for elderly populations in tertiary care settings, highlighting the significant burden of AF in aging societies. While modifiable risk factors such as hypertension, diabetes, obesity, and CAD were significantly associated with AF, non-modifiable contributors like age and possible genetic predisposition also play a critical role in its pathogenesis. Given the high frequency of asymptomatic or atypical presentations in older adults, routine ECG screening - preferably conducted annually or opportunistically - in individuals aged 70-79 with comorbidities is recommended. Such screening, particularly in primary care and community-based settings, has shown cost-effectiveness in reducing stroke risk and enabling early diagnosis. AF screening and prevention efforts should be actively integrated into public health programs and geriatric care models. Additionally, there is a pressing need for future longitudinal research to develop and evaluate more effective, age-specific preventive strategies, tailored to the unique risk profile and clinical presentation of the elderly population.

## References

[1] Chugh SS, Roth GA, Gillum RF, Mensah GA (2014). Global burden of atrial fibrillation in developed and developing nations. Glob Heart.

[2] Hindricks G, Potpara T, Dagres N (2021). 2020 ESC Guidelines for the diagnosis and management of atrial fibrillation developed in collaboration with the European Association for Cardio-Thoracic Surgery (EACTS): the task force for the diagnosis and management of atrial fibrillation of the European Society of Cardiology (ESC) developed with the special contribution of the European Heart Rhythm Association (EHRA) of the ESC. Eur Heart J.

[3] Benjamin EJ, Wolf PA, D'Agostino RB, Silbershatz H, Kannel WB, Levy D (1998). Impact of atrial fibrillation on the risk of death: the Framingham Heart Study. Circulation.

[4] Go AS, Hylek EM, Phillips KA, Chang Y, Henault LE, Selby JV, Singer DE (2001). Prevalence of diagnosed atrial fibrillation in adults: national implications for rhythm management and stroke prevention: the anticoagulation and risk factors in atrial fibrillation (ATRIA) study. JAMA.

[5] Psaty BM, Manolio TA, Kuller LH (1997). Incidence of and risk factors for atrial fibrillation in older adults. Circulation.

[6] Boriani G, Laroche C, Diemberger I (2015). Asymptomatic atrial fibrillation. Am J Med.

[7] Halcox JP, Wareham K, Cardew A, Gilmore M, Barry JP, Phillips C, Gravenor MB (2017). Assessment of remote heart rhythm sampling using the Alivecor heart monitor to screen for atrial fibrillation: the REHEARSE-AF study. Circulation.

[8] Patton KK, Zacks ES, Chang JY, Shea MA, Ruskin JN, Estes NA (2004). Clinical features and outcomes of atrial fibrillation patients aged <60 years. Am J Cardiol.

[9] Siontis KC, Gersh BJ, Killian JM (2016). Typical, atypical, and asymptomatic presentations of new-onset atrial fibrillation in the community: characteristics and prognostic implications. Heart Rhythm.

[10] Benjamin EJ, Levy D, Vaziri SM, D'Agostino RB, Belanger AJ, Wolf PA (1994). Independent risk factors for atrial fibrillation in a population-based cohort. The Framingham Heart Study. JAMA.

[11] Huxley RR, Lopez FL, Folsom AR (2011). Absolute and attributable risks of atrial fibrillation in relation to optimal and borderline risk factors: the Atherosclerosis Risk in Communities (ARIC) study. Circulation.

[12] Movahed MR, Hashemzadeh M, Jamal MM (2005). Diabetes mellitus as a risk for atrial fibrillation. Int J Cardiol.

[13] Al Chekakie MO, Welles CC, Metoyer R (2010). Pericardial fat is independently associated with human atrial fibrillation. J Am Coll Cardiol.

[14] Bansal N, Fan D, Hsu CY, Ordonez JD, Marcus GM, Go AS (2013). Incident atrial fibrillation and risk of end-stage renal disease in adults with chronic kidney disease. Circulation.

[15] Zimmerman D, Sood MM, Rigatto C, Holden RM, Hiremath S, Clase CM (2014). Systematic review and meta-analysis of incidence, prevalence and outcomes of atrial fibrillation in patients on dialysis. Nephrol Dial Transplant.

[16] Wolf PA, Abbott RD, Kannel WB (1991). Atrial fibrillation as an independent risk factor for stroke: the Framingham Study. Stroke.

[17] Granger CB, Alexander JH, McMurray JJ (2011). Apixaban versus warfarin in patients with atrial fibrillation. N Engl J Med.

[18] Freedman B, Camm J, Calkins H (2017). Screening for atrial fibrillation: a report of the AF-SCREEN international collaboration. Circulation.

[19] Pathak RK, Middeldorp ME, Meredith M (2015). Long-term effect of goal-directed weight management in an atrial fibrillation cohort: a long-term follow-up study (LEGACY). J Am Coll Cardiol.

[20] Abed HS, Wittert GA, Leong DP (2013). Effect of weight reduction and cardiometabolic risk factor management on symptom burden and severity in patients with atrial fibrillation: a randomized clinical trial. JAMA.

[21] Nielsen JB, Graff C, Pietersen A (2013). J-shaped association between QTc interval duration and the risk of atrial fibrillation: results from the Copenhagen ECG study. J Am Coll Cardiol.

